# A high bandwidth dimension ratio compact super wide band-flower slotted microstrip patch antenna for millimeter wireless applications

**DOI:** 10.1016/j.heliyon.2023.e23712

**Published:** 2023-12-16

**Authors:** V N Koteswara Rao Devana, A. Beno, Mohammed S. Alzaidi, P. Bala Murali Krishna, G. Divyamrutha, Wahaj Abbas Awan, Thamer A.H. Alghamdi, Moath Alathbah

**Affiliations:** a ECE Department, Aditya Engineering College, Surampalem, Kakinada, A.P, India; b ECE Department, Dr. Sivanthi Aditanar College of Engineering, Tiruchendur, India; c Department of Electrical Engineering, College of Engineering, Taif University, P.O. Box 11099, Taif 21944, Saudi Arabia; d ECE Department, Chalapathi Institute of Technology, Guntur, India; e ECE Department, Malla Reddy Engineering College, Maisammaguda, Hyderabad, Telangana, India; f Department of Information and Communications Engineering, Chungbuk National University, Cheongju 28644, South Korea; g Wolfson Centre for Magnetics, School of Engineering, Cardiff University, Cardiff CF24 3AA, UK; h Electrical Engineering Department, School of Engineering, Albaha University, Albaha 65779, Saudi Arabia; i Department of Electrical Engineering, College of Engineering, King Saud University, Riyadh 11451, Saudi Arabia

**Keywords:** BDR, BR, Millimeter wave, RB-DGS, SWB, Taper

## Abstract

A compact high bandwidth ratio (BDR) super wide band flower slotted micro strip patch antenna (SWB-FSMPA) for super wide band (SWB) applications is presented. The SWB-FSMPA is constructed on a FR-4 substrate having a size of 16 × 22 mm^2^. The SWB-FSMPA incorporates a 50 Ω tapered micro strip line and a rectangular beveled defected ground structure (RB-DGS). This design enables a simulation bandwidth from 3.78 to 109.86 GHz, allowing for coverage of various wireless applications such as WiMAX (3.3–3.6 GHz), 5G (3.3–3.7 GHz), WLAN (5.15–5.825 GHz), UWB (3.1–10.6 GHz), Ku– (12–18 GHz), K– (18–27 GHz), Ka– (27–40 GHz), V– (40–75 GHz), and W– (75–110 GHz) millimeter wave bands. The SWB-FSMPA antenna exhibits a gain that varies within the range of 3.22–7.23 dBi and a peak efficiency of 93.3 %. The SWB-FSMPA possesses a bandwidth ratio (BR) of 29.1:1, a BDR of 5284 in the frequency domain, a minimal group delay (GD) fluctuation of <0.48 ns, and a linear phase in the time domain, making it well-suited for SWB applications.

## 1 Introduction

In wireless communication applications, the monopole antenna plays a vital role because of their simplicity in design, ease of integration to other components and more importantly higher bandwidths. Recently, for short- and long-range communications, it is needed to enhance the spectrum [1]. The design of the SWB antenna offers a viable approach to enhancing bandwidth. While no precise frequency range is designated for SWB technology, it is generally accepted that the radiator has BR of 10:1 or more is considered as a SWB radiator, irrespective of its frequency band. The key challenges in designing of SWB antennas are vary depending upon the application it is intended. Firstly, the millimeter wave antenna requires extremely high bandwidths in 5G communications for achieving higher data rates [2]. Secondly, the design of compact size antennas for millimeter wave applications to cover super wide bandwidth can be a challenge. Thirdly, achieving adequate gain and efficiency over the entire super wide band is crucial. Thus, antenna designers need to optimize the radiation pattern and efficiency to ensure effective communication or sensing. A nature inspired sunflower shaped antenna with theoretical analysis that covers Ku band used for electromagnetic community with improved radiation performance in satellite communications is reported [3,4]. In literature [[5], [6], [7], [8], [9], [10], [11], [12]], so many monopole antennas were investigated to operate for 1–2 GHz for L, S 2–4 GHz for S, 4–8 GHz for C, 8–12 GHz for X, 12–18 GHz for Ku, 18–27 GHz for K, 27–40 GHz for Ka and 40–75 GHz for V band applications. In Ref. [5], a novel monopole radiator to cover a narrow bandwidth of 1.42–2.52 GHz is reported for GPS/PCS/Bluetooth applications with a BR of 1.77:1. A monopole antenna with a horizontal slot along with a dual slot inset feed is reported to achieve a bandwidth of 1 GHz with resonance at 4.28 GHz operating over 3.25–4.25 GHz with BR of 1.3:1 for *C*-band applications [6]. A beveled monopole antenna with DGS is operated over 2.75–11.05 GHz for UWB, and X band applications is presented in Ref. [7] having a BR of 4.04:1. A monopole element of size of 16 × 26 mm^2^ with a bandwidth of 3.1–18.8 GHz to operate for S, C, X, and Ku bands having a BR of 6.06:1 is investigated in Ref. [8]. A novel lamp slotted patch with semicircular ground structure having a size of 16 × 22 mm^2^ to cover UWB, X, & Ku bands over 3.63–21.94 GHz with a BR of 6.04:1 is proposed in Ref. [9]. A tapered fed compact SE-DGS monopole antenna having 15 × 18 mm^2^ size has an operating bandwidth of 4.11–29.77 GHz is reported in Ref. [10] having a BR of 7.24:1 operating for UWB, X, Ku and K bands. A SWB radiator using a meander line, a quarter waveguide transformer feed, and a DGS with a size of 35 × 35 mm^2^ operating at 3.08–40.9 GHz to cover UWB, X, Ku, K and Ka bands with a BR of 13.27:1 is reported in Ref. [11]. A very high BR of 32.2:1 monopole SWB-CSF antenna having 24 × 30 mm^2^ size, operating over 2.99–95.82 GHz to cover S, C, X, Ku, K, Ka and V band applications is reported in Ref. [12]. From the challenges of designing of millimeter wave antennas as discussed and from literature [[1], [2], [3], [4], [5], [6], [7], [8]], the proposed work is focused to design a SWB antenna for millimeter wave application to cover a very wide bandwidth, a compact sized radiator, and an acceptable gain and efficiency over the entire operating band is crucial. The millimeter technology has the potential to deliver extremely high data rates, which is vital for applications such as, high definition video streaming, mobile broadband and emerging technologies like virtual and augmented reality [13,14]. Millimeter-wave radar and sensing systems are critical for autonomous vehicles. These systems rely on wideband antennas to provide high-resolution imaging and accurate object detection, contributing to the safety and effectiveness of self-driving cars [15]. Millimeter wave frequencies are valuable for imaging and sensing applications, including security scanners, medical imaging, and industrial sensing. Super-wideband antennas can enhance the resolution and accuracy of these systems. The Internet of Things (IoT) and Industry 4.0 are driving the need for reliable and high-capacity wireless communication in industrial settings. Millimeter wave technology can enable real-time data exchange in smart factories, and wideband antennas are essential for robust connectivity. Beyond 5G, there is ongoing research into future wireless technologies, such as 6G. These technologies are expected to push the boundaries of data rates and applications even further, necessitating advanced millimeter wave antenna designs. Millimeter wave technology continues to find new and innovative applications in areas like healthcare, environmental monitoring, and agriculture. Wideband antennas play a vital role in enabling these applications to meet their performance requirements. Thus, millimeter wave antennas are essential for achieving high data rates, enabling new technologies, and addressing the demands of modern wireless communication and sensing systems.

This paper details the design and fabrication of a monopole SWB-FSMPA with a RB-DGS, which is able to transmit signals from 3.78 to 109.8 GHz. The merits of the SWB-FSMPA are: a tiny size of (16 × 22 mm^2^), a novel flower slot structure, a wide spectrum of 3.78–109.86 GHz, fractional bandwidth of 186 %, BR of 29.1:1, BDR of 5284, peak efficiency of 93.3 %, GD of <0.48 ns, and a linear type phase that proves to be a better contender for millimeter wave applications [16].

## 2 Antenna design

**Fig. 1:**
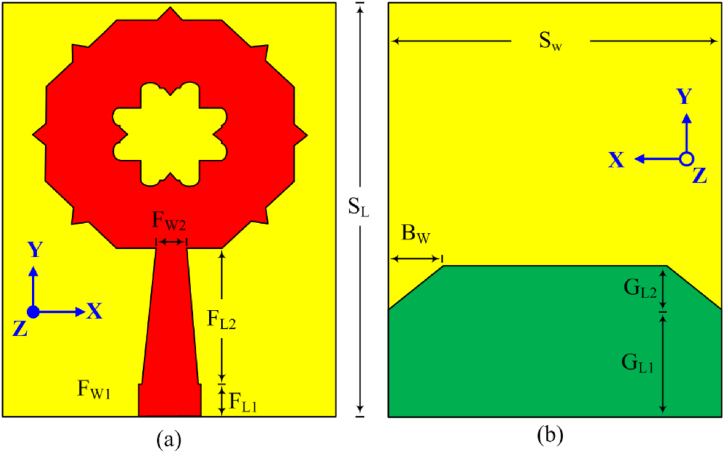
Geometry of SWB-FSMPA (a) front view (b) back view.

**Fig. 2:**
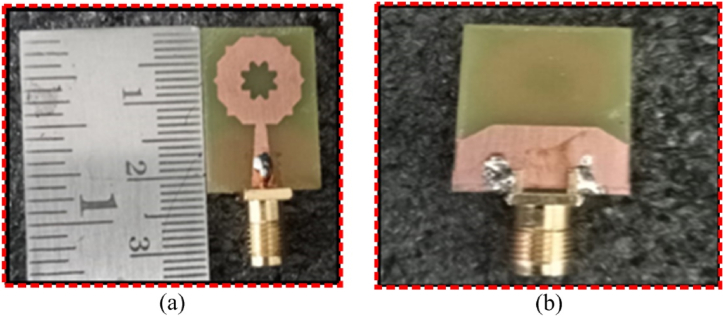
SWB-FSMPA prototype (a) front view (b) back view.

An inexpensive FR-4 substrate of size, S_W_ × S_L_ mm^2^, permittivity, ε_r_ = 4.3, thickness = 1.6 mm, and loss tangent, δ = 0.02 are used to print the suggested SWB-FSMPA. The geometry and fabricated prototype of SWB-FSMPA is delineated in Fig. 1(a and b) and Fig. 2(a and b), respectively. The FSMPA top layer comprises of a flower slotted circular type of structure with a micro strip line of F_W1_ × F_L1_ mm^2^ and is tapered by F_W2_ × F_L2_ mm^2^, and a RB-DGS on the other side of substrate. The RB-DGS has a total length of G_L1_+G_L2_ mm and is made up of two bevel-type slots of B_W_ × G_L2_ mm^2^ at the corners. The optimized parameters of SWB-FSMPA are (in mm): S_W_ = 16, S_L_ = 22, F_W1_ = 3, F_W2_ = 1, F_L1_ = 1, F_L2_ = 10, G_L1_ = 5.75, G_L2_ = 2.4, B_W_ = 3.

## 3 Simulation results

This section details the systematic approach used to create an SWB-FSMPA with a modified patch, ground, and feed structures.

### 3.1 Implementation of SWB-FSMPA patch structure

**Fig. 3:**
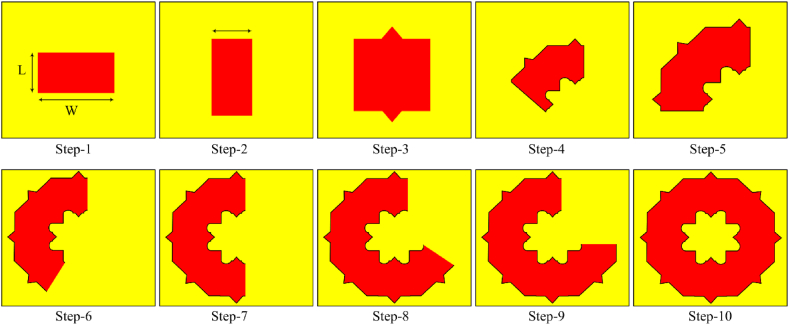
Implementation of FSMPA patch.

The implementation stages of proposed SWB-FSMPA patch structure is described in Fig. 3. The patch of proposed antenna structure is obtained by the intersection of a rectangular shaped element with a 45° rotated square shaped structure and then the achieved structure is repeated eight times with a rotation angle of 45° as depicted in Fig. 3. Then, the final antenna structure of proposed antenna is optimized in CST Software to achieve the desired wideband impedance bandwidth characteristics. In design and development of the proposed antenna, the antenna is designed based on the rectangular/square shaped patch antenna design as given in Ref. [17]. Also, introducing slots in the patch structures and connected regions increases the bandwidth of the antenna [18]. Firstly, the patch of SWB-FSMPA is designed with intersection angle of 45° of a rectangle of W × L mm^2^ with a square of side L mm structure as represented in Fig. 3 (Step 1–2). In the next stage (Step-3), the structure obtained is transformed eight times with a rotation angle of 45° to achieve the proposed SWB-FSMPA structure as shown in Fig. 3 (Step 4–10), respectively.

### 3.2 Parametric study with varying ground and feed

**Fig. 4:**
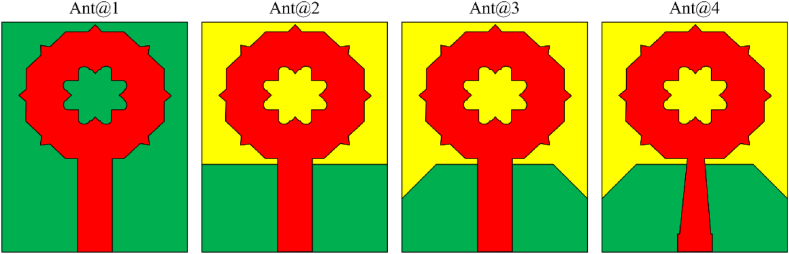
Evolution stages of FSMPA with ground and feed structures.

**Fig. 5:**
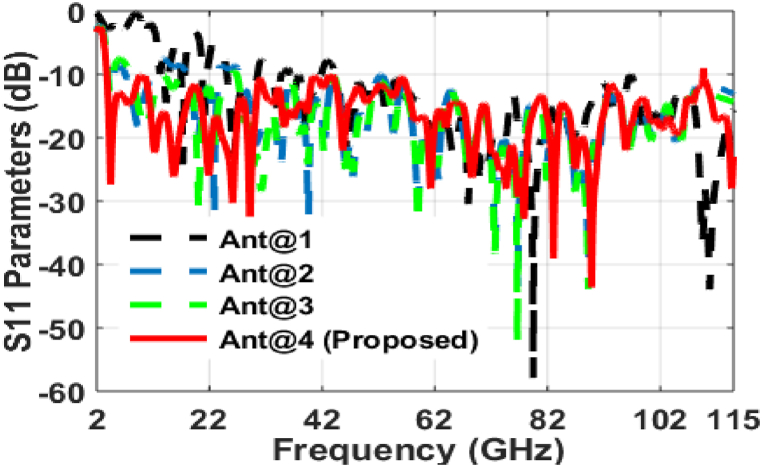
S_11_ plot for DGS and feed line variations of FSMPA.

Fig. 4 displays how the ground and feed structures of FSMPA affects the performance of the radiator at various stages. The structure Ant@1, with full ground structure, the FSMPA produces a narrow bands at 13.98–14.8 GHz, 16.98–17.65 GHz, 20.73–21.55 GHz, 25.08–30.55 GHz, 32.98–34.74 GHz, 36.28–41.84 GHz, and 44.48–96.24 GHz with resonant frequencies 14.3 GHz, 17.3 GHz, 21.04 GHz, 26.27 GHz, 34.02 GHz, 39.26 GHz, and 79.82 GHz, respectively with peak of S_11_ of −14.49 dB, −24.23 dB, −17.39 dB, −23.13 dB, −15.24 dB, −19.36 dB, and −58.24 dB, respectively as in Fig. 5. With the rectangular DGS structure, Ant@2, the FSMPA generates resonances at 4.38 GHz, 9.34 GHz, 22.87 GHz, and 39.53 GHz with peak of S_11_ of −12.94 dB, −18.31 dB, −36.13 dB, and −36.05 dB, respectively over 3.98–5.12 GHz, 7.28–11.64 GHz, 18.05–23.56 GHz, and 27.27 to more than 110 GHz, respectively as in Fig. 5. Introducing DGS on the ground plane can help in suppressing unwanted modes and improving impedance matching, which can lead to wider bandwidth [19,20]. For the RB-DGS structure, Ant@3 as represented in Fig. 4, the FSMPA having peak S_11_ values of −12.88 dB, −31.38 dB, and −51.84 dB over a spectrum of 3.93–4.94 GHz, 7.94–25.94 GHz, and 26.82 to more than 110 GHz, respectively with resonant frequencies at 4.31 GHz, 20.08 GHz, and 76.84 GHz, respectively as depicted in Fig. 5. Gradually tapering the ground plane away from the patch antenna can reduce edge effects and improve bandwidth [21]. Finally, with proposed tapered structure Ant@4 of FSMPA as delineated in Fig. 4, the antenna produces a spectrum of 3.78–109.86 GHz with peak S_11_ of −43.62 dB at 89.8 GHz as depicted in Fig. 5. Thus, the tapered micro strip line feed increases the bandwidth of FSMPA over a wide bandwidth as in Ref. [22].

## 4 Results and discussion

### 4.1 Frequency domain analysis

**Fig. 6:**
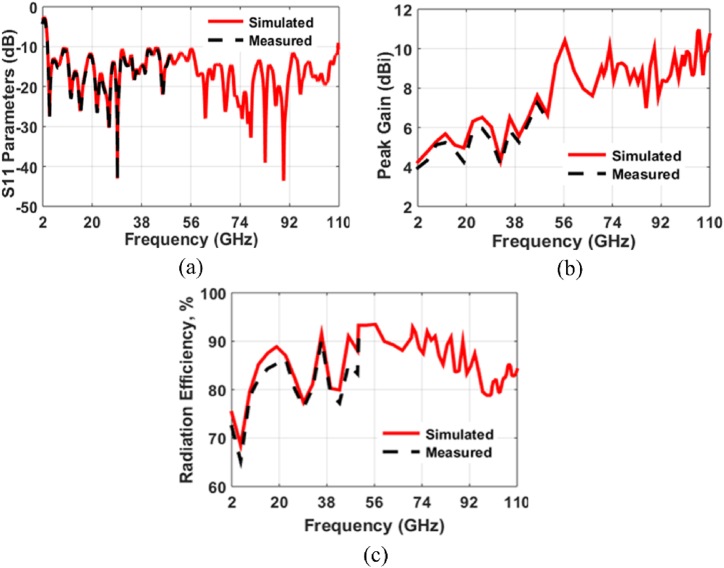
Characteristics of SWB-FSMPA (a) S_11_ (b) gain (c) efficiency.

**Fig. 7:**
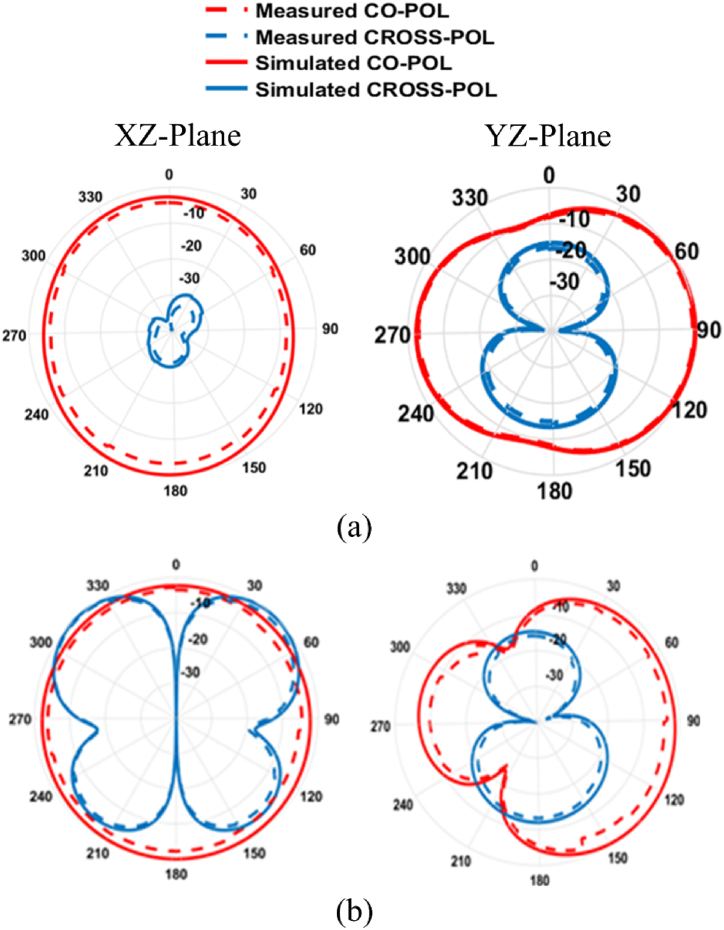
Radiation pattern of the proposed work at (a) 4.44 GHz (b) 29.2 GHz.

**Fig. 8:**
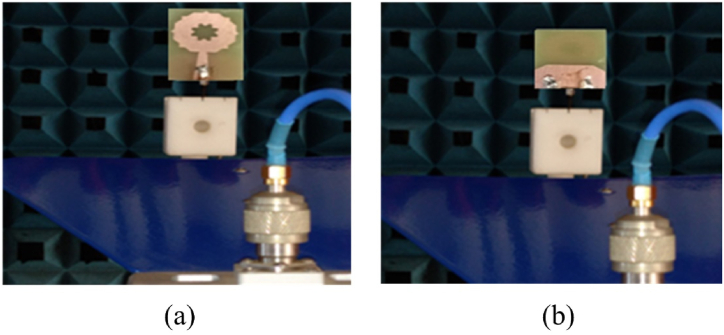
Measurement setup in anechoic chamber (a) front view (b) back view.

The SWB-FSMPAs electrical properties are evaluated using an Anritsu MS2037C/2 vector network analyzer, which measures parameters including return loss (S_11_), peak gain, and efficiency. Analyzing the differences between the calculated and observed *S*-parameters yielded the findings shown in Fig. 6 (a). The actual curve exhibits modest disparities in comparison to the simulated curve due to factors such as fabrication precision and potential variations in the welding of SMA joints. The measured bandwidth is 3.78 to more than 50 GHz as depicted in Fig. 6(a). Fig. 6(b and c) illustrates the radiator's measured gain and efficiency. The radiator has a gain ranging from 4.23 to 7.12 dBi. The antenna's efficiency across the working frequency ranges from 68.87 % to 90.23 %. Fig. 7(a and b) depicts the observed and simulated patterns of the SWB-FSMPA in E and H plane at 4.44 GHz and 29.2 GHz, respectively. Radiation patterns in E-plane are bidirectional, whereas those in H-plane are almost omnidirectional. Whereas Fig. 8(a and b) shows the measurements setup for far-field parameters.

To determine BDR by Ref. [23],

(1) $$BDR=\frac{\%\;FBW}{\lambda_{Length}\lambda_{Width}}$$

Where, $\lambda_{Length}$ and $\lambda_{Width}$ are the electrical length and width of radiator respectively, and λ is wavelength at lower cut off frequency. According to Equation (1), more bandwidth (3.78–109.86 GHz) and shorter electrical wavelength (0.16λ × 0.22λ) lead to higher BDR (5284) values of SWB-FSMPA. Table 1 shows the BDR values of several wide band radiators compared to the SWB-FSMPA, making it evident that the suggested SWB-FSMPA possesses the larger BDR value. This demonstrates that the anticipated SWB-FSMPA is state-of-the-art in comparison to other radiator designs in use currently, both in terms of bandwidth and size.

**Table 1:** Comparison parameters of SWB-FSMPA with literature.

Ref.	Size (mm^2^)	Area (mm^2^)	Electrical size	Bandwidth (GHz)	FBW (%)	BR	BDR	Efficiency (%)
[24]	74 × 80	5920	0.26λ × 0.28λ	1.05–32.7	187	31:1	2586	–
[25]	60 × 60	3600	0.23λ × 0.33λ	2.18–44.5	181	20.4:1	2461	95
[26]	60 × 60	3600	2λ × 2 λ	10–50	133	5:1	33	–
[27]	35 × 77	2695	0.17λ × 0.37λ	1.44–18.8	172	13:1	2735	–
[28]	50 × 50	2500	0.35λ × 0.35λ	2.1–11.6	138	5.5:1	1126	88
[29]	38 × 55	2090	0.38λ × 0.55λ	3–35	168	11.6:1	803	–
[30]	30 × 45	1350	0.31λ × 0.47λ	3.15–32	164	10.1:1	1101	–
[31]	32 × 32	1024	0.31λ × 0.31λ	2.9–15	135	5.17:1	1404	79.21
[32]	25 × 35	875	0.29λ × 0.4λ	3.5–31.9	160	9.14:1	1379	–
[33]	31 × 28	868	0.31λ × 0.28λ	3–12.8	124	4.26:1	1428	–
**Prop.**	**16** × **22**	**352**	**0.16λ** × **0.22λ**	**3.78**–**109.86**	**186**	**29.1:1**	**5284**	**93.3**

### 4.2 Time domain analysis

**Fig. 9:**
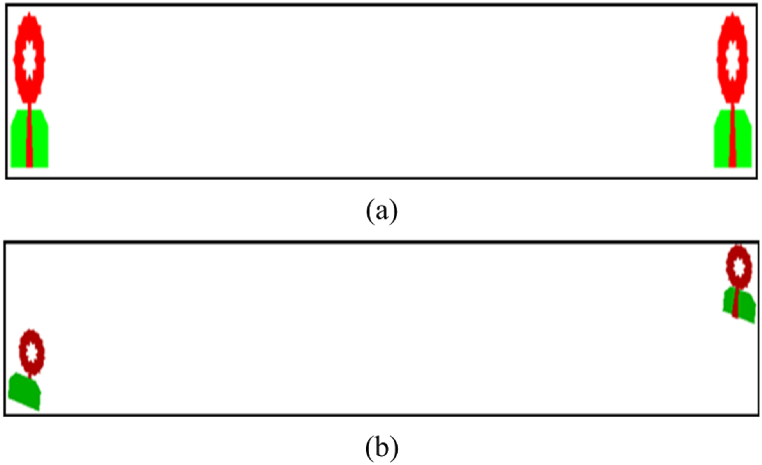
Setup for time domain analysis (a) Side-by-side (b) Face-to-face.

**Fig. 10:**
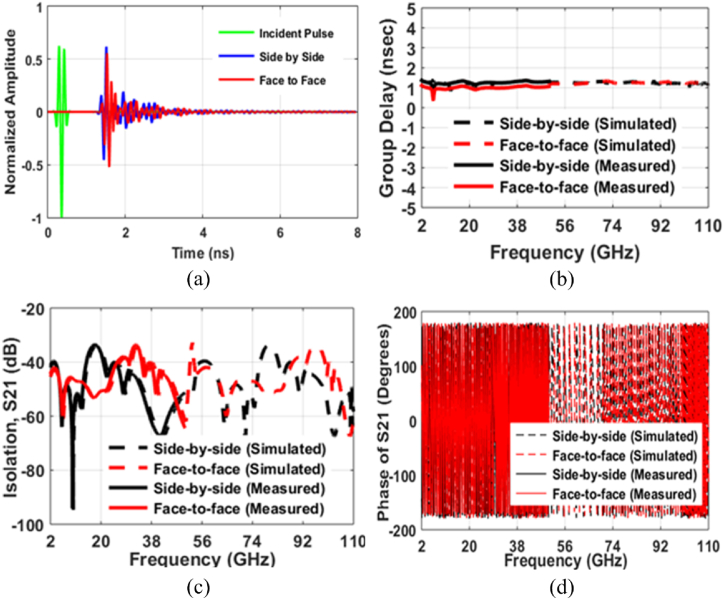
Time domain characteristics comparison among simulated and measured (a) Transmitted and received pulses (b) Group Delay (c) S_21_ (d) Phase.

Time domain study of a SWB-FSMPA is equally crucial for validity as frequency domain analysis. The SWB-FSMPA's time-domain analysis incorporates two antenna sections spaced 30 cm apart as transceivers in both typical face-to-face and side-by-side architectures as in Fig. 9(a and b). The normalized pulse amplitudes that were transmitted and received in both topologies appear in Fig. 10(a). The group delay for both setups is displayed in Fig. 10(b). The GD varies by just 0.48 ns from the 3.78–50 GHz range in both systems. Thus, the fluctuations in GD < 1 ns of proposed SWB-FSMPA confirms minimum pulse distortion as the phases in far-field are linear. The isolation between two SWB-FSMPAs is illustrated in Fig. 10(c). A significant level of isolation of < -35 dB guarantees the transmission of uncorrelated signals on both ports. In Fig. 10(d), the phase of S_21_ is shown as a function of frequency for the SWB-FSMPA in both configurations. The abundance of linear phase deviations in both topologies implies the dearth of any divergent features in the signal that was acquired. Hence, it can be deduced that the time domain characteristics mentioned above provide insights into the transmission qualities related to minimum pulse distortion.

The performance evaluation of the SWB-FSMPA was carried and the results are shown in Table 1, which is in agreement with the existing literature. A modified rectangular patch with an asymmetric trapezoid ground plane having a size of 74 × 80 mm^2^, covering a bandwidth of 1.05–32.7 GHz with BR of 31:1 and BDR of 2586 is reported in Ref. [24]. In Ref. [25], a circular patch with iterations of a hexagonal slot with a size of 60 × 60 mm^2^, spanning a spectrum of 2.18–44.5 GHz, BR of 20.4:1, BDR of 2461 and peak efficiency of 95 % is proposed. An octagonal fractal patch antenna can be utilized for 10–50 GHz having a size of 60 × 60 mm^2^, with a BR of 5:1 and BDR of 33 is presented in Ref. [26]. A planar microstrip fed semi elliptically fractal complementary slot embedded into the asymmetrical ground plane having 35 × 77 mm^2^ size, covering a spectrum of 1.44–18.8 GHz with BR of 13:1, and BDR of 2735 is reported in Ref. [27]. A transmission line loaded square slot antenna having 50 × 50 mm^2^ size, to achieve an UWB response over 2.6–11.5 GHz with a BR of 5.5:1, a BDR of 1126 and peak radiation efficiency of 88 % is proposed in Ref. [28]. A CPW-fed circular disk to resemble a propeller shaped monopole antenna occupying 3–35 GHz bandwidth with a BR of 11.6:1, BDR of 803 and having a dimension of 38 × 55 mm^2^ is presented in Ref. [29]. A rectangular radiator equipped with staircase technique and mounting two major and minor rectangular symmetrical stubs on top of the quarter circle slot ground by dual axis to increase the bandwidth over 3.15–32 GHz having a size of 30 × 45 mm^2^, with a BR of 10.1:1, and a BDR of 1101 is recommended in Ref. [30]. Several iterations of pentagonal slot inscribed in a circular structure fractal antenna with DGS having impedance bandwidth over 2.9–15 GHz with a compact size of 30 × 45 mm^2^ having 5.17:1 of BR, 1404 of BDR, and efficiency of 79.21 % is proposed in Ref. [31]. A circular disc monopole antenna for current and future UWB applications is reported [32] with a compact size of 25 × 35 mm^2^ with bandwidth of 3.5–31.9 GHz, BR of 9.14:1 and BDR of 1379. By etching a Koch fractal geometry in patch as well in the ground plane of the antenna demonstrated in Ref. [33] can produce a spectrum over 3–12.8 with a BR of 4.26:1, BDR of 1428 and having a compact dimension of 31 × 28 mm^2^. In comparison to the reported antennas in literature [[24], [25], [26], [27], [28], [29], [30], [31], [32], [33]], the proposed antenna is having the following merits: produce a very wide bandwidth of 3.78–109.8 GHz, having a compact size of (16 × 22 mm^2^), a novel flower slot structure, fractional bandwidth of 186 %, the bandwidth ratio (BR) of 29.1:1, the Bandwidth Dimension Ratio (BDR) of 5284, the peak efficiency of 93.3 %, and in time domain, the group delay of (GD) of <0.48 ns, and a linear type phase that proves to be a better contender for millimeter wave applications.

## 5 Conclusion

This work proposed a novel design of a tiny SWB-FSMPA with a high BDR and SWB capabilities. The antenna incorporates flower-shaped slot to achieve enhanced performance for SWB applications. The proposed SWB-FSMPA facilitates a simulation bandwidth that spans from 3.78 to 109.86 GHz, thereby accommodating a wide range of wireless applications including UWB, Ku, K, Ka, V, and W band millimeter wave bands. The SWB-FSMPA radiator demonstrates a variable gain ranging from 3.22 to 7.23 dBi, with a maximum efficiency of 93.3 %. The SWB-FSMPA has a BR of 29.1:1, a frequency domain BDR of 5284, a low variation in group delay (GD) of less than 0.48 ns, and a linear phase in time domain. These characteristics make it highly suitable for SWB applications. A super wide band antenna design at millimeter-wave frequencies is more challenging and often requires sophisticated techniques and technologies. Small wavelengths demand precise manufacturing tolerances and high-quality materials.

## Additional information

No additional information is available for this paper.

## Data availability statement

Data included in article/supp. material/referenced in article.

## CRediT authorship contribution statement

**V N Koteswara Rao Devana:** Writing – original draft, Software, Resources, Conceptualization. **A. Beno:** Writing – original draft, Software, Resources, Methodology, Investigation, Formal analysis, Data curation. **Mohammed S. Alzaidi:** Writing – original draft, Software, Resources, Conceptualization. **P. Bala Murali Krishna:** Writing – original draft, Methodology, Investigation, Formal analysis, Data curation. **G. Divyamrutha:** Writing – original draft, Methodology, Investigation, Formal analysis, Data curation. **Wahaj Abbas Awan:** Writing – review & editing, Validation, Supervision, Funding acquisition. **Thamer A.H. Alghamdi:** Writing – review & editing, Visualization, Validation, Project administration, Funding acquisition. **Moath Alathbah:** Writing – review & editing, Visualization, Validation.

## Declaration of competing interest

The authors declare that they have no known competing financial interests or personal relationships that could have appeared to influence the work reported in this paper.
